# Gambling Disorder and Romantic Relationships: The role of positive communication and emotional dysregulation in couple satisfaction

**DOI:** 10.1007/s10899-025-10410-1

**Published:** 2025-06-30

**Authors:** Laura Macía, Irati Saratxaga, Alexander Álvarez-González, Ioseba Iraurgui, Ana Estévez

**Affiliations:** Psychology Department, School of Health Sciences, University of Deusto, Av. de las universidades 24, Bilbao, 48007 Spain

**Keywords:** Gambling Disorder, Couple Relationships, Positive Communication, Couple Satisfaction, Emotional Dysregulation

## Abstract

Although gambling disorder (GD) may affect romantic relationships, and these may influence its prevention and intervention, there is a gap about this issue in the literature. People with GD present greater emotional dysregulation, and lack of communication and couple satisfaction is observed in their romantic relationships. Therefore, the objectives of the present study were, first, to explore the differences between couple satisfaction, positive communication and emotional dysregulation in individuals with GD and their partners. Second, to analyse the relationships between the variables in GD patients, their partners, and betwen the couple members. Third, to study the predictive role of positive communication and emotional dysregulation in couple satisfaction. The sample comprised 30 people: 15 male individuals with gambling disorder and their respective female partners. Results showed that the partners scored lower in couple satisfaction. Likewise, positive correlations were found between communication and satisfaction, but only the partners’ emotional dysregulation significantly correlated with the aforementioned variables. Finally, only positive communication predicted couple satisfaction. Consequently, it is argued that communication and emotion-regulation skills in romantic relationships could influence the development, maintenance, and rehabilitation of GD. Providing comprehensive care that enhances romantic relationships could be beneficial because both individuals can reciprocally influence each other’s mental health.

What can cause previously satisfying romantic relationships to become sources of frustration? (Ross et al., 2022). Behavioural theories such as the social learning theory (Bandura, 1977) or the social exchange theory (Thibaut & Kelley, 1959) have been applied to the field of romantic relationships (Jacobson & Margolin, 1979; Markman, 1979), with communication as a fundamental basis for their evolution (Kelly et al., 2003). These models agree that the quality of communication can affect members’ subjective evaluation of their relationship (Johnson et al., 2022); that is, their satisfaction (Fincham & Rogge, 2010) or the degree to which the couple manifests intimacy, affection and mutual support (Collins et al., 2009).

## Relationship and Gambling Disorder

Gambling disorder is defined in the *Diagnostic and statistical manual of mental disorders* (5th ed. [DSM-5-TR], American Psychiatric Association, [APA], 2022) as a non-substance related addictive disorder. It consists of persistent and recurrent problematic gambling behaviour that causes clinically significant impairment or distress (APA, 2022) (see Annex 1). Of the people admitted to treatment for gambling disorder in Spain in 2021, 28.7% suffered from financial problems, 27.5% from family conflicts, 16% from mental and physical health problems, 8.6% from work problems and 7.1% from loss of family relationships such as divorce, separation or loss of custody (Observatorio Español de las Drogas y las Adicciones [Spanish Observatory of Drugs and Addictions], 2023). After economic problems, family conflicts are the most common consequence. However, the effects of gambling disorder on the family and its impact on family intervention have hardly been analysed (Kourgiantakis et al., 2013).

Cunha and Relvas (2014) propose an integrative systemic model to understand gambling disorder, considering the following levels: the social context, the family, the couple dynamics and the individual. It focuses on the couple, the first and most affected subsystem (Lee, 2002), with two assumptions about gambling behaviour: (a) it has a determinant impact on the subsystem, influenced by previous difficulties in the subsystem, and (b) it may be a sign of previous problems in the subsystem (Cunha & Relvas, 2014).

Regarding the association between gambling disorder and the family, a meta-analysis by Kourgiantakis et al. (2013) yields two lines of research: family involvement in treatment can improve individual and family functioning, and gambling disorder has consequences on individual family members and on family dynamics. In relation to gamblers’ partners, three ideas emerge: partners are unaware of or misunderstand the problem, they experience the consequences of gambling individually, in the family and socially, and these consequences can be counteracted by adaptive coping strategies. Riley et al. (2021) conducted a meta-synthesis on gamblers’ partners, finding that, compared to the general population, they had more mental and physical health problems and higher tobacco and alcohol consumption. Concerning the impact on the relationship, interpersonal conflict, divorce and separation stand out. Alarmingly, female partners of males with gambling disorder were more frequently victims of gender-based violence than the general population.

The gender bias in the literature reviewed is worth highlighting, as most research focuses on heterosexual couples in which the men have a diagnosis of gambling disorder (Dowling et al., 2009; Ponti et al., 2021). In the study by Estévez et al. (2023), female gamblers reported an overload of family responsibilities focused on caregiving, making their rehabilitation difficult. Some of the participants had lost the support of their partners, others ended the relationship as it exacerbated the problem, and partner abuse was a recurrent theme. Therefore, one would expect gambling disorder to have a different impact on heterosexual couples with female versus male gamblers.

### Lack of Communication

Couple satisfaction increases when reinforcing interactions are experienced and decreases when interactions extinguish or punish positive behaviours (Kelly et al., 2003). That is, positive communication patterns increase satisfaction, and negative communication patterns erode satisfaction (Johnson et al., 2022). Positive communication is characterised by reasoning, problem-solving, and showing positive affect and support. In contrast, negative communication involves belittling behaviours, blaming and showing negative affect (Overall et al., 2009).

It has been found that partners of gamblers may perceive loneliness in their relationship. Klevan et al. (2019) found that loneliness was associated with taking on financial and family responsibilities that created inequality in the relationship and a feeling of not being able to ‘burden’ their partner during the treatment period. Similarly, Riley et al. (2020) found that the partners perceived disconnection, reporting parallel lives despite living together, as they lacked emotional and physical intimacy and claimed to be taking on the ‘mother role’, changing the relational dynamics and losing their partner’s support. Finally, these relationship difficulties have also been associated with a lack of trust due to lies (Holdsworth et al., 2013), with deception and a sense of dishonesty being a frequent theme (Tepperman et al., 2006).

### Couple Satisfaction

Concerning satisfaction in romantic relationships, Ponti et al. (2021) compared couples in which one partner had a diagnosis of a gambling disorder with control couples. Compared to the control group, gamblers perceived more conflict and their partner’s less trust in the relationship and less willingness to help, while their partners expressed less emotional connection and relationship quality. In the study by Jeffrey et al. (2019), gamblers reported a lack of accountability in their relationships, and their partners were five to six times more likely to perceive greater tension and conflict and to end the relationship.

In turn, both partners in the relationship have shown lower dyadic adjustment than the general population both in couples with male gamblers (Fernandez et al., 2002) or female gamblers (Dowling et al., 2009), and greater severity of the gambling disorder has been associated with lower dyadic adjustment (Tepperman et al., 2006). However, in the study by Fernandez et al. (2002), female partners reported significantly lower satisfaction compared to gamblers. Furthermore, Cunha and Relvas (2015) found that people with gambling disorder showed a similar level of marital adjustment and satisfaction as the general population, but their partners scored lower on the satisfaction and consensus dimensions of dyadic adjustment, as well as on roles, communication and conflict, and relationship continuity. It is worth noting that while both partners may experience a worsening of their relationship as a consequence of gambling (Tepperman, 2006), Cunha and Relvas’ (2015) study did not consider gambling as the main family problem.

### The Role of Emotional Dysregulation

On the one hand, gamblers have been found to manifest greater emotional dysregulation than the general population in cross-sectional studies (Jauregui et al., 2016; Williams et al., 2012) and meta-analyses (Neophytou et al., 2023; Velotti et al., 2021). Gratz and Roemer (2004) defined emotional dysregulation as difficulties in the dimensions of: (a) emotional awareness and understanding, (b) emotional acceptance, (c) adopting goal-directed behaviours and refraining from impulsive behaviours when experiencing unpleasant emotions, and (d) the ability to flexibly employ emotion-regulation strategies, modulating emotional responses according to situational demand and individual goals. Mestre-Bach et al. (2020) observed that difficulties lie in emotional identification as well as in the selection and implementation of emotion-regulation strategies. Therefore, the study of emotional dysregulation represents a central component of treatment and rehabilitation.

On the other hand, regarding the psychological impact of addiction on the partners of gamblers, it has been found that gamblers manifest anger, resentment and depression (Holdsworth et al., 2013; Lorenz & Yaffee, 1988), suicidal thoughts, confusion, hopelessness and perceived parental ineffectiveness (Lorenz & Yaffee, 1988), stress and anxiety (Holdsworth et al., 2013), and guilt and responsibility for the addiction (Holdsworth et al., 2013; Lorenz & Yaffee, 1988). In addition, emotional dysregulation has been associated with lower couple satisfaction (Rick et al., 2017; Sousa-Gomes et al., 2023; Velotti et al., 2016; Xu et al., 2023) and with the perception that partners communicate negatively (Klein et al., 2016).

### The Role of the Partner in Treatment

Lee (2002) used congruence couples therapy, finding that gamblers and their partners sought intimacy and reconnection but that their communication was characterised by a lack of trust and respect, criticism, avoidance and ridicule, as well as unexpressed thoughts, feelings, expectations and desires. Fifty per cent of partners reported verbal, emotional and/or physical abuse at some point in their relationship. Similarly, Tremblay et al. (2015) recommended integrative couples therapy for the treatment of gambling disorder, whose goals are to reduce or stop gambling behaviour and increase well-being, couple satisfaction and mutual support. Communication skills are a central aspect, and it is assumed that increasing satisfaction will decrease the likelihood of relapse.

Finally, Côté et al. (2020) explored the impact of gamblers‘ partners’ coping strategies on gambling behaviour. When faced with conflict, gamblers reported that their partners’ maladaptive strategies generated unpleasant emotions that they regulated by gambling, whereas adaptive strategies would help their rehabilitation. In addition, fear of losing their relationships was the most useful mechanism for achieving abstinence.

However, although the literature points to the impact of addiction both on partners and individuals with gambling disorderand the possible impact of gambling on couple satisfaction, few studies have explored this issue (Holdsworth et al., 2013; Kourgiantakis et al., 2013; Lorenz & Yaffee, 1988; Riley et al., 2021). Similarly, few studies have explored the impact of gambling on the couple relationship (Fernandez et al., 2002; Jeffrey et al., 2019; Ponti et al., 2021; Tepperman et al., 2006), or the influence of the couple relationship both on the triggering of gambling behaviour (Côté et al., 2020; Cunha & Relvas, 2014) and rehabilitation (Lee, 2002; Tremblay et al., 2018).

## Objectives

Therefore, the first aim of the research is to study the differences in the three variables (positive communication, emotional dysregulation and couple satisfaction) between the two partners in the relationship (the individuals with gambling disorder and the partners). The next objectives are to study the relationship between positive communication, emotional dysregulation and couple satisfaction in the person with gambling disorder (second objective), and in the partner (third objective). The fourth objective is to explore the relationship of each variable both in individuals with gambling disorderand partners. Finally, the fifth aim analyses the predictive role of positive communication and emotional dysregulation in the couple satisfaction of individuals with gambling disorder and in the case of the partner (sixth objective).

### Hypotheses

Regarding the hypotheses, for the first objective, partners are expected to score lower in satisfaction, and individuals with gambling disorder to score higher in emotional dysregulation. Regarding the second and third objectives, both gambling disorder patients and partners are expected to show positive correlations between positive communication and satisfaction, and negative correlations between emotional dysregulation and positive communication and satisfaction. As for the fourth objective, positive correlations of the variables between individuals with gambling disorderand partners are expected, except for the correlations between emotional dysregulation and positive communication and satisfaction, which would be negative. Finally, positive communication and emotional dysregulation are expected to predict couple satisfaction both in gambling disorder patients and partners (fifth and sixth objectives, respectively).

## Method

### Participants

Due to difficulty in locating the participants, this was a convenience sample, comprising a total of 30 people, 15 male diagnosed with a gambling disorder, and the other 15 were their respective female partners. The participants are or have been attended to in associations for the rehabilitation of gambling disorder in Spain. The inclusion criteria are as follows: (a) being of legal age, (b) maintaining a romantic relationship lasting at least six months at the time of assessment (Sánchez-Fuentes et al., 2015), and (c) men with gambling disorder must be receiving or have received treatment for gambling disorder. Specifically, in these associations, the treatment for gambling disorder is group-based and non-inpatient. In the case of the accompanying partners, they are offered the possibility of attending family group treatment. Occasionally, systematic or couple interventions can be carried out. These systemic interventions are especially aimed at counselling the partner on how to deal with monetary control and other specific issues to support abstinence.

Regarding the characteristics of the sample, participants were 15 male individuals with gambling disorder (50%; hereafter “individuals with gambling disorder”) and 15 female partners (50%; hereafter “partners”). The gambling disorder patients’ age was between 21 and 67 years (*M* = 42.20, *SD* = 14.11) and the partners’s age ranged between 19 and 65 years (*M* = 39.67, *SD* = 13.12). Twenty-nine people (96.7%) were of Spanish nationality and one person was of non-Spanish nationality (3.3%).

Concerning marital status, six couples were married (40%), eight couples had a common-law marriage (53.3%), and one were an unmarried couple (6.7%). The duration of the relationship ranged from nine months to 50 years. Regarding cohabitation, 12 couples (80%) were cohabiting, and three couples (20%) were not, with the duration of cohabitation ranging from nine months to 42 years. Seven couples (46.7%) had children, and eight couples (53.3%) did not. Finally, of the seven couples who had children, three couples had one child (20%), three couples had two children (20%), and one couple had three children (6.7%).

As shown in Table 1, the individuals with gambling disorder had a family history of gambling disorder and drug addiction. Regarding psychological or psychiatric antecedents, individuals with gambling disorder presented cannabis use disorder and obsessive-compulsive disorder. Concerning the partners, four had been diagnosed with depression, one with depression and anxiety and another also with bipolar disorder.

**Table 1:** Sociodemographic analysis of individuals with gambling disorder and their partners

Sociodemographic variables		% Total	% GD patients	% Partners
Level of education	No education	3.3	6.7	
	Primary education	10	6.7	13.3
	Compulsory secondary education	13.3	26.7	
	High school	16.7	13.3	20
	Vocational training	26.7	33.3	20
	University studies	30	13.3	46.7
Employment status	Working	83.3	86.7	80
	Unemployed	3.3		6.7
	Studying	3.3		6.7
	Retired	6.7	13.3	
	Other	3.3		6.7
Family antecedents of	Yes	20	20	0
addictions	No	80	80	100
Psychological	Yes	23.3	13.3	26.7
antecedents	No	76.7	86.7	73.3

## Instruments

### Sociodemographic Data

Participants were asked to identify as a person with gambling disorder or a partner, age, biological sex, gender identity, nationality, treatment phase (first, second or third phase) or treatment completion, level of education completed, employment status, marital status, family history of gambling or other addictions, psychological or psychiatric history, duration of their relationship, duration of cohabitation and number of children. They were assigned an identification code (described below).

### Emotional Dysregulation

To measure emotional dysregulation, the Spanish version of the Difficulties in Emotional Regulation Scale (DERS: Hervás & Jódar, 2008), based on the original DERS scale developed by Gratz and Roemer (2004), was used. It comprises 28 items and 5 subscales: Emotional Discontrol (9 items), Emotional Rejection (7 items), Everyday Interference (4 items), Emotional Inattention (4 items) and Emotional Confusion (4 items). Examples of items are: ‘I experience my emotions as overflowing and out of control’ (Emotional Discontrol) or ‘When I feel bad, I am ashamed to feel that way’ (Emotional Rejection). Responses are rated on a five-point Likert scale ranging from 1 (*almost never*) to 5 (*almost always*). The higher the score, the more emotional dysregulation the person manifests. In the study by Hervás and Jódar (2008), the total scale obtained an internal consistency of 0.93, 0.91 for Dyscontrol, 0.90 for rejection, 0.87 for Interference, 0.73 for Inattention and 0.78 for Confusion. Test-retest reliability was adequate, and convergent and incremental validity were good. The present study obtained a high reliability with a Cronbach’s alpha of 0.93.

### Satisfaction in the Couple

The Satisfaction with Couple Relationship Scale (SCR: Urbano-Contreras et al., 2017) was applied to measure couple satisfaction. The scale contains 10 items rated on a four-point Likert scale, ranging from 1 (*strongly disagree*) to 4 (*strongly agree*). The higher the score, the higher the satisfaction with the relationship. Examples of items are: ‘I feel valued by my partner’ and ‘My partner shows me the affection and love I need’. Urbano-Contreras et al. (2017) obtained an internal consistency of 0.93, suggesting that the instrument is suitable for use in empirical contexts due to its brevity and easy application. In the present study, Cronbach’s alpha was 0.93, a high internal consistency.

### Communication in the Couple

To measure communication in the couple’s relationship, we used the Self-perceived Communication in the Couple Relationship Scale (CARP: Iglesias-García et al., 2019). It comprises 8 items and 2 subscales: Positive Communication (4 items) and Negative Communication (4 items). Positive communication is characterised by expressing feelings and thoughts, affection, cooperation, understanding and respect, whereas negative communication is defined by a conflictive, oppressive and disrespectful attitude, devaluing the partner’s experience (Sánchez-Aragón & Díaz Loving, 2003). Examples of items are: ‘When something about my partner bothers me, I inform him/her, respecting his/her point of view’ (positive communication) and ‘I tend to tell my partner the negative things I see in him/her before the positive ones’ (negative communication). It is rated on a four-point Likert scale ranging from 1 (*strongly disagree*) to 4 (*strongly agree*). Iglesias-García et al. (2019) obtained an internal consistency of.75 for the total scale,.79 for the Positive Communication subscale and.73 for the Negative Communication subscale, values between good and acceptable. Convergent validity was good for positive communication and moderate for negative communication. The authors suggest that the scale is suitable for use in empirical contexts due to its psychometric properties and brevity. In the present study, Cronbach’s alpha was.53.

### Procedure

The sample was obtained through associations for the reahabilitation of gambling disorder between April and July 2024. First of all, information leaflets were distributed with a brief summary of the participation procedure and the general objectives of the research to justify its importance. For this purpose, the leaflets were distributed to the Gambling Disorder Mutual Aid Groups and the Mutual Aid Group for family members, as well as informing the rehabilitated volunteers.

All participants gave their informed consent to participate anonymously. They were informed about the scientific purpose of the research and their right to leave the study at any time and the confidentiality of their data. An identification code, known only to the couple, was used to link the questionnaires of the two members of the couple anonymously—the initial of the individual´s with gambling disorder first surname and day of birth, and then the initial of the partner’s first surname and day of birth. They did not receive any reward for their collaboration.

Participants could self-administer the online or paper version of the questionnaire, which took approximately 15 min to complete. The questionnaire included general information about the study’s main objectives and contact information to allow them to receive further information.

The research was approved by the ethics committee of the University of Deusto (ref.: ETK-37/23–24) and was conducted following the principles of the Helsinki Declaration.

### Data Analysis

This was a cross-sectional correlational study. Statistical analyses were conducted using SPSS 27.0 (IBM Corp., 2020). Descriptive analyses were carried out to obtain a sociodemographic sample description.

Firstly, for the first objective, mean differences between the group of people with gambling disorder and the group of female partners were analysed using Student’s t-test for related samples. Secondly, Pearson’s correlation coefficients were calculated to assess the relationships between the study variables, thus exploring the second, third and fourth objectives. Thirdly, hierarchical linear regressions were performed to analyse the predictive role of positive communication and emotional dysregulation on couple satisfaction. For this purpose, a three-step model was analysed, introducing positive communication in the first step, emotional dysregulation in the second step, and the interaction between the two variables in the third step. Regression analysis was performed on individuals with gambling disorder and partners to analyse the fifth and sixth objectives.

## Results

First, mean differences in emotional dysregulation, couple satisfaction and positive communication were analysed between the group of individuals with gambling disorder and the partner group (Table 2). The partner group scored significantly lower on satisfaction than the group of individuals with gambling disorder. No significant differences were obtained in the rest of the study variables.

**Table 2:** Difference in means between the group of individuals with gambling disorder and the group of partners

	GD group	Partners group		
	*M* (S*D*)	*M* (*SD*)	*t*(*df*)	*p*
1. Emotional dysregulation	64.13(20.01)	63.13(22.65)	0.12(14)	0.906
2. Satisfaction	36.27(5.79)	32.73(8.32)	2.64(14)	0.019*
3. Communication	4.80(3.59)	5.53(4.58)	-0.72(14)	0.480

**p <.01. *p <.05

Secondly, the correlations between the study variables were analysed (Table 3). In the case of the group of individuals with gambling disorder, their satisfaction positively correlated with their positive communication, but emotional dysregulation did not correlate significantly with any variable. In the case of the partner group, their satisfaction correlated positively with their positive communication and negatively with their emotional dysregulation. Likewise, positive communication and satisfaction correlated positively and significantly both in the partner group and the group of individuals with gambling disorder. Finally, partners’ emotional dysregulation correlated negatively with individuals´ with gambling disorder, and positive with communication and satisfaction, whereas individuals` with gambling disorder emotional dysregulation did not correlate significantly with any of the partners’ variables.

**Table 3:** Correlation coefficients between satisfaction, communication and emotional dysregulation in individuals with gambling disorder and partners

	1	2	3	4	5	6
1. Individuals with gambling disorder Satisfaction	-					
2. Individuals with gambling disorder Communication	0.75**	-				
3. Individuals with gambling disorder Emotional Deregulation	0.02	0.26	-			
4. Partner Satisfaction	0.79**	0.78**	0.21	-		
5. Partner Communication	0.62*	0.56*	0.17	0.72**	-	
6. Partner Emotional Dysregulation	− 0.76**	− 0.71**	− 0.14	− 0.65**	− 0.49	-

**p <.01. * *p* <.05

Thirdly, the predictive role of positive communication and emotional dysregulation inindividuals’ with gambling disorder and partners’ satisfaction was analysed (Tables 4 and 5). Positive communication was the only variable predicting satisfaction for both.

**Table 4:** Hierarchical multiple regression of communication and emotional dysregulation on individuals’ with gambling disorder satisfaction

	t	B	SEB	β	F(*p*)	*R*	*R*²	adj. *R*²	Change in *R*²
*Step 1*					16.59(0.001)*	0.75	0.56	0.53	0.56
communication	4.07*	1.21	0.30	0.75					
*Step 2*					0.91(0.359)	0.77	0.59	0.53	0.03
communication	4.17*	1.28	0.31	0.80					
dysregulation	− 0.95	− 0.05	0.06	− 0.18					
*Step 3*					2.79(0.123)	0.82	0.67	0.59	0.08
communication	4.52	1.30	0.29	0.81					
dysregulation	− 0.63	− 0.03	0.05	− 0.12					
Interaction	-1.67	− 0.04	0.02	− 0.30					

*p <.05

**Table 5:** Hierarchical multiple regression of communication and emotional dysregulation on partner’s satisfaction

	t	B	SEB	β	F(*p*)	*R*	*R*²	adj.*R*²	Change in *R*²
*Step 1*					13.84(0.003)*	0.72	0.52	0.48	0.52
Partner communication	3.72*	1.30	0.35	0.72					
*Step 2*					3.70(0.078)	0.75	0.63	0.57	0.11
Partner communication	2.61*	0.96	0.37	0.53					
Partner dysregulation	− 0.19	− 0.14	0.07	− 0.39					
*Step 3*					0.196(0.666)	0.80	0.64	0.54	0.006
Partner communication	1.99	0.86	0.43	0.48					
Partner dysregulation	-1.54	− 0.13	0.08	− 0.35					
Interaction	0.44	0.01	0.01	0.11					

*p <.05

## Discussion

The first aim of this study was to analyse differences in emotional dysregulation, positive communication and couple satisfaction between individuals with gambling disorder and their partners. We found that partners scored significantly lower on couple satisfaction than individuals with gambling disorder, as previous studies on gambling disorder and couple relationships have found (Cunha & Relvas, 2015; Fernandez et al., 2002; Jeffrey et al., 2019; Ponti et al., 2021). Partners of gamblers have been found to perceive less companionship, security and intimacy in their relationships than the general population (Ponti et al., 2021), and to experience the consequences of addiction more intensely in their romantic relationships than gamblers, with greater awareness of dysfunctionality (Jeffrey et al., 2019). Klevan et al. (2019) found that gamblers’ partners suffered stigma and shame for remaining in the relationship and coped alone with responsibilities such as organisation, finances and emotional support.

In the present study, individuals with gambling disorder are or have been accompanied in the therapeutic process by their partners, while the latter have faced the consequences of addiction and may have assumed parallel responsibilities such as financial control or emotional care. Therefore, on the basis of the results, we hypothesise that the individuals´ with gambling disorder satisfaction with their partners may have increased as they felt supported, while their partners’ satisfaction may have decreased due to the imbalance in roles and the greater awareness of the difficulties in the relationship. It should be noted that, although the results may have been affected by gender bias because all individuals with gambling disorder were men and all partners were women, Jackson et al. (2014) indicated that the differences reflected in the literature, with women reporting lower couple satisfaction than men, may be limited to people who have sought couple counselling. However, it is also worth mentioning that people with gambling disorder in this sample were receiving group psychological treatment, so this issue may also have influenced the results.

Likewise, no significant differences were found in emotional dysregulation. Individuals with gambling disorder were expected to score significantly higher on this variable (Jauregui et al., 2016; Neophytou et al., 2023; Velotti et al., 2021; Williams et al., 2012). However, emotional dysregulation is not unique to gambling disorder but is a transdiagnostic dimension of psychological disorders such as anxiety or depression (Carmassi et al., 2022). Gamblers’ partners may present anxious-depressive symptoms such as stress, sleep problems, difficulty concentrating, irritability, loss of appetite, anhedonia, etc. (Holdsworth et al., 2013; Lorenz & Yaffee, 1988; Tepperman et al., 2006) and may also score high on emotional dysregulation. In the present study, four of the fifteen people in the partner group (26.7%) had a diagnosis of depression, which could influence the results.

Concerning the second and third objectives, the relationship between the study variables was analysed in each couple member. The results show that positive communication was positively correlated with couple satisfaction in both individuals with gambling disorder and partners, coinciding with Woodin (2011). Contrary to our expectations, emotional dysregulation did not correlate with positive communication in either member. However, emotional regulation was positively correlated with perceived constructive communication (Bloch et al., 2014), and emotional dysregulation predicted perceived negative partner communication (Klein et al., 2016). Furthermore, partners’ emotional dysregulation was negatively correlated with couple satisfaction, although this was not the case for individuals with gambling disorder. While emotional dysregulation is associated with satisfaction for both couple members (Sousa-Gomes et al., 2023), differential gender emotional socialisation could explain these results. People are educated according to their gender to play different emotional roles in their relationships, influencing their emotion-regulation skills: emotional responsibility is attributed to women and avoidance of emotional expression and ‘rationality’ to men (Suberviola Ovejas, 2020). That is, women are socialised to be oriented towards romantic relationships and manage them emotionally, whereas men may be less effective in dealing with highly emotional situations in interpersonal contexts (Bloch et al., 2014). Therefore, when presenting emotional dysregulation, the partners may feel emotionally overburdened by the gender role. In addition, in their search for support, they may run into the individuals´ with gambling disorder lack of emotional skills.

Regarding the fourth objective, the relationship between variables was analysed, this time between individuals with gambling disorder and partners. Individuals´ with gambling disorder and partners’ positive communication and couple satisfaction correlated positively, in line with Kanter et al. (2022). Whereas individuals´ with gambling disorder emotional dysregulation did not correlate with any variable, partners’ emotional dysregulation correlated negatively with positive communication and satisfaction of people with gambling disorder.

The non-significant results in individuals´ with gambling disorder emotional dysregulation could indicate the importance of exploring each dimension of the construct and attending to context, especially when dealing with couples in high-stress situations (Rick et al., 2017). For example, counterintuitively, the emotional rejection dimension of the DERS has previously been associated with higher short-term couple satisfaction (Rick et al., 2017; Xu et al., 2023). As each dimension of the DERS could be associated in different ways with satisfaction, unifying them may have affected the results.

In contrast, in the study by Bloch et al. (2014), only women’s emotional regulation predicted increases in their own and in men’s satisfaction. Moreover, only women’s constructive communication longitudinally revealed the association between their emotional regulation and satisfaction. Along the same lines, women’s emotional dysregulation has been found to be more relevant than men’s in both members’ satisfaction (Rick et al., 2017; Xu et al., 2023). That is, women could act as emotional regulators in heterosexual couples (Bloch et al., 2014), explaining the results obtained and aligning with studies that include gender perspective.

However, when interpreting the results of this study, we should also consider the structural aspect of the research, in which gender differentiates at all times the role of ‘partner’ (women) or ‘individual with gambling disorder’ (men). Therefore, we cannot claim that it is the effect of gender, but that it could be the “role” of the partner that causes these results. Consequently, the differences observed in variables such as emotional dysregulation cannot be attributed exclusively to “gender” or to “the partner role”, as it is not possible to statistically dissociate them in this study. This limitation has to be taken into account when interpreting the results, and future studies could include samples of women with gambling disorders and their male partners, including same-sex couples.

Finally, positive communication and emotional dysregulation were expected to predict couple satisfaction in individuals with gambling disorder (fifth objective) and partners (sixth objective). The results are mixed, as positive communication was the only predictor in both cases. On the one hand, the communication findings align with the literature: communication patterns play an essential role in theories on the evolution of romantic relationships because of their strong influence on couple satisfaction (Kelly et al., 2003). In Williamson’s (2021) longitudinal study, couples with poorer communication divorced more than twice as often as those with better communication. That is, communication may act as a protective factor. On the other hand, there is evidence that emotional dysregulation may predict couple satisfaction. Specifically, emotional dysregulation has been associated with poorer relationship quality in terms of intimacy, predicting fear of emotional involvement and difficulties in perceiving a partner’s emotions (Tani et al., 2015), as well as the satisfaction of both couple members (Xu et al., 2023). Emotional regulation is therefore considered to be a protective factor.

In the case of gambling disorder, gamblers may resort to gambling as a way of emotionally regulating themselves after a conflict with their partner (Hagen et al., 2023, Côté et al., 2020). According to Côté et al. (2020) emotional dysregulation and communication skills of both couple members play an important role in the maintenance of gambling behaviour. Gamblers reported that conflict and their partner’s maladaptive strategies generated negative emotions, precipitating their gambling behaviour to regulate themselves emotionally. In contrast, a partner’s positive communication could help to reduce gambling behaviour emotionally.

In summary, training in emotion regulation and positive communication to manage couple conflict could help to maintain gambling abstinence and prevent relapse (Hagen et al., 2023). In addition, several studies have applied couple therapy to intervene in gambling disorder. After therapy, couples reported more frequent expression of their thoughts, feelings and desires, as well as decreased negative communication patterns, and they perceived greater connection, security and intimacy (Lee, 2002). Similarly, Tremblay et al. (2018) concluded that both couple members wanted to understand gambling and the effects of gambling on their partner. They also indicated that couple therapy improved communication, and the partner contributed to motivation to change, adherence to treatment and relapse prevention.

### Limitations and Future Studies

The present study is not without limitations, the main one being the sample size, which has had several consequences. On the one hand, the DERS and CARP subscales were not analysed due to statistical power. The CARP is an instrument with two subscales with positive and negative items whose internal consistency may have been affected by unifying them (α = 0.53). Furthermore, Johnson et al. (2022) have suggested that negative communication has a greater effect on couple satisfaction. In the present study, only positive communication was analysed. We also think that the relevance of emotional dysregulation would have been reflected with a larger sample and by analysing the DERS subscales (Rick et al., 2017). On the other hand, some of the individuals with gambling disorder were rehabilitated, while others were still in treatment. The participants attended or had attended associative centres, and there may be differential characteristics with other clinical samples, such as people who have not sought help or who attend private centres or public hospitals. Therefore, the heterogeneity of the sample in terms of the different stages of treatment in which the participants are involved should be mentioned as an important limitation. Future studies may benefit from subgroup analyses according to stage of treatment. In addition, it would also be beneficial to measure the variables before and after treatment in both partners. In addition, female partners may have also participated in the Mutual Help Group of family members.

Another limitation lies in the cross-sectional nature of the study, making it impossible to interpret the causality and directionality of the effects. Similarly, self-report instruments may affect the validity of the results due to social desirability, among other aspects. Finally, the gender bias stands out, as the results might be different if the sample consisted of heterosexual couples with female gamblers, due to factors such as lack of social support, family care overload or violence (Estévez et al., 2023).

As for possible future challenges, the main one would be to overcome the gender bias and analyse heterosexual couples with female gamblers, as well as homosexual couples. Although the literature shows that sexual and gender minorities may present greater psychological vulnerability, there are hardly any studies on LGBT people diagnosed with gambling disorder (Lee & Grubbs, 2023). Similarly, other assessment methods could be used to complement self-report instruments (e.g., a specific emotional dysregulation instrument for the couple context), compare clinical and general populations (Rick et al., 2017) and analyse negative communication. Other variables to be considered are stress, as it may moderate the association between communication and couple satisfaction (Nguyen et al., 2020), and dyadic coping (the couple’s joint coping capacity in the face of stress) because it may mediate the association between emotional intelligence and satisfaction (Zeidner et al., 2013). In addition, relationship duration (Mazzuca et al., 2019), or socioeconomic status (Ross et al., 2019), among others, should be taken into account. On the other hand, it should also be mentioned that gambling disorder was not assessed in female partners through a questionnaire. Future studies could benefit from assessing gambling in both partners, as recent studies such as that of Suomi et al. (2024) stated that intimate partners are more vulnerable to gambling-related harms, and Estévez et al. (2023), found that women are more likely to initiate gambling through their partners.

Finally, future research could benefit from incorporating a qualitative component to deepen the understanding of the interpersonal dynamics between individuals with gambling disorder and their partners. Qualitative methods may help capture the complexity of relational experiences, emotional processes, and the role of intimate bonds in both relapse and recovery. This approach could complement quantitative findings and provide a more comprehensive view of the couple’s experience.

## Conclusions

Despite the limitations, the study has multiple strengths and implications, the main one being the exploration of gambling disorder and couple relationships, an area that has been little examined. The present study involved both individuals with gambling disorder and their partners, in contrast to studies on couple satisfaction, where often only one partner has been included (Jackson et al., 2014). Moreover, it is a quantitative analysis and can complement previous qualitative studies. Finally, evidence is provided for positive communication as a possible protective factor for couple satisfaction in relationships affected by gambling disorder. Similarly, partners’ emotional dysregulation has also been negatively correlated with communication and individuals with gambling disorder ’ satisfaction and may also act as a protective factor. The importance of exploring protective factors in addition to risk factors is highlighted.

The literature shows that gambling disorder does not only harm the individual, but the people close to them also suffer the consequences of the addiction. Hence, we propose an approach that considers gambling disorder from a systemic perspective, taking into account the individual but also the partner, the family, and the context. As couple relationships are the most affected subsystem, this may be the most urgent intervention area. On the one hand, gamblers’ partners experience serious consequences of the gambling disorder, mainly at the psychological and relational levels. This may be exacerbated in the case of female partners in heterosexual relationships due to gender roles. On the other hand, couple dynamics, and specifically communication and emotional regulation skills associated with conflict, may influence the development, maintenance and rehabilitation of addiction. In short, the results suggest that the variables should be studied in a larger sample, to provide evidence on the importance of offering comprehensive and networked care, with dyad work being relevant, as both couple members in the relationship can influence each other’s mental health.

## Data Availability

The datasets generated during and/or analysed during the current study are not publicly available due to confidentially.
